# Exploring Chemical Diversity Alongside Antioxidant, Antimicrobial, Antiproliferative, Anti-Inflammatory, and *Caenorhabditis elegans* Locomotor Responses in Whole, Caps and Stipes of *Suillus luteus* and *Agaricus bisporus*

**DOI:** 10.3390/ph19101571

**Published:** 2026-10-03

**Authors:** Elena Coyago-Cruz, Juan A. Puente-Pineda, Rebeca Gonzalez-Pastor, Julieth Soledispa, Angélica Geovanna Zea Cobos, Oscar Carrillo-Naranjo, Carlos Barba-Ostria, Jorge Heredia-Moya, Gina Tafur

**Affiliations:** 1 Carrera de Ingeniería en Biotecnología, Universidad Politécnica Salesiana, Sede Quito, Campus El Girón, Av. 12 de Octubre N2422 y Wilson, Quito 170143, Ecuador; 2 Centro de Investigación Biomédica (CENBIO), Facultad de Ciencias de la Salud Eugenio Espejo, Universidad UTE, Quito 170527, Ecuador; 3 Grupo de Innovación Educativa, Seguridad y Soberanía Alimentaria (GIE-SYSA), Universidad Politécnica Salesiana, Sede Cuenca, Av. Turuhuayco 3-69 y Calle Vieja, Cuenca 010150, Ecuador; 4 Escuela de Medicina, Colegio de Ciencias de la Salud Quito, Universidad San Francisco de Quito (USFQ), Quito 170901, Ecuador; 5 Instituto de Microbiología, Universidad San Francisco de Quito (USFQ), Quito 170901, Ecuador; 6 Carrera de Ingeniería Agropecuaria, Universidad Politécnica Salesiana, Sede Quito, Campus Cayambe, Av. Natalia Jarrín N3-85 y 9 de Octubre, Cayambe 170501, Ecuador

**Keywords:** phenolics, edible mushrooms, antiproliferative effects

## Abstract

Edible mushrooms contain chemically diverse metabolites that can be characterised for their biological response under experimental conditions. This study characterised whole mushrooms, including caps and stipes, from wild *Suillus luteus* and cultured *Agaricus bisporus* var. *brunnescens*, and evaluated their physicochemical composition, bioactive compounds, and antioxidant, antimicrobial, antiproliferative, anti-inflammatory, and locomotor activities in *Caenorhabditis elegans*. The extracts were evaluated using spectrophotometric, chromatographic, ABTS, and DPPH assays, as well as microdilution, MTT, nitrite production in RAW 264.7 macrophages, and locomotor parameters. *Agaricus bisporus* var. *brunnescens* contained 46.7 and 50.2 mg/100 g of vitamin C in the whole mushroom and the cap, respectively, and between 1685.9 and 2152.8 mg/100 g DW of total phenols. *S. luteus* contained 38,609.0–51,974.6 mg/100 g DW of total organic acids, primarily malic acid, and exhibited higher antioxidant capacity ranging from 3.2 to 3.3 mmol TE/100 g DW as measured by the DPPH assay and 4.9–5.9 as measured by the ABTS assay. *A. bisporus* showed growth-inhibitory activity against all eight bacterial strains tested and exhibited the lowest MIC against *Staphylococcus aureus* (3.9 mg/mL), whereas no inhibition was detected against the two *Candida* strains tested. *S. luteus* showed an IC_50_ of 0.08–0.21 mg/mL, although with limited tumour selectivity, and reduced LPS-induced nitrite levels by up to 64.6%. The locomotor changes observed in *C. elegans* were not statistically significant. These results are exploratory and serve as a starting point for future research.

## 1. Introduction

Edible fungi have become increasingly important in the human diet due to their nutritional value and their potential as a source of bioactive compounds with health-promoting effects. This interest is part of the search for functional foods that can help prevent chronic non-communicable diseases, whose incidence continues to rise worldwide. Among the pathophysiological mechanisms involved in these diseases, oxidative stress plays a key role, as it results from an imbalance between the production of reactive oxygen species (ROS) and the capacity of endogenous antioxidant systems to neutralise them, causing cellular damage, metabolic alterations and tissue dysfunction [1,2]. Consequently, identifying food matrices rich in compounds that modulate these processes is a priority in the fields of nutrition, biomedicine, and nutraceuticals [3].

In this context, edible fungi represent a particularly attractive alternative as they combine high nutritional value with a wide diversity of secondary metabolites. Since ancient times, various cultures have used fungi not only as food but also as a medicinal resource associated with health and longevity [4,5,6]. They are currently recognised as functional foods due to their high-quality protein content, which includes all essential amino acids, as well as their low fat and significant dietary fibre content, particularly chitin and β-glucans [4]. They also constitute an important source of essential micronutrients, notably potassium, phosphorus, iron and selenium, as well as B-complex vitamins and vitamin D precursors [7].

Beyond their nutritional properties, mushrooms are recognised for containing numerous bioactive compounds, including phenolic compounds, flavonoids, carotenoids, organic acids, antioxidant vitamins, terpenoids and bioactive polysaccharides. These metabolites are involved in various biological mechanisms related to cellular protection against oxidative damage, regulation of inflammatory processes, and modulation of immune responses [7,8]. In particular, phenolic compounds and carotenoids possess a remarkable ability to neutralise free radicals and reduce cellular oxidative processes, thereby helping prevent diseases associated with oxidative stress [2,8]. This capacity is typically assessed using widely accepted methods, such as the ABTS and DPPH assays, which allow estimation of the antioxidant potential of natural food extracts and matrices [7].

Scientific interest in edible fungi has also increased due to their antimicrobial properties. The growing spread of microorganisms resistant to conventional antimicrobials has driven the search for new bioactive molecules of natural origin with therapeutic potential [9]. Various studies have shown that compounds present in mushrooms, such as phenols, tannins, terpenoids and other secondary metabolites, can disrupt the integrity of microbial cell membranes, inhibit essential enzymatic systems and interfere with nucleic acid synthesis [7]. As a result, species in genera such as *Agaricus* and *Pleurotus* have shown activity against clinically significant Gram-positive and Gram-negative bacteria, including *Staphylococcus aureus*, *Bacillus subtilis*, *Escherichia coli*, and *Pseudomonas aeruginosa* [7,9]. Furthermore, some fungal extracts have been reported to enhance the efficacy of conventional antibiotics through synergistic effects, representing a promising strategy in the face of the global challenge of antimicrobial resistance [9].

Furthermore, various compounds present in fungi have demonstrated complementary biological activities related to the prevention and control of chronic diseases. Among these, β-glucans, phenolic compounds and terpenoids have been associated with mechanisms of action that include modulation of the immune system, regulation of inflammatory processes, induction of apoptosis and inhibition of abnormal cell proliferation [10]. These properties have established edible fungi as one of the most extensively studied natural sources in the search for new functional ingredients and nutraceuticals.

Despite the growing body of research on edible fungi, most studies have focused on specific aspects, such as evaluating a single biological activity or characterising isolated groups of bioactive compounds. Therefore, the aim of this study was to comprehensively characterise two species of edible mushrooms, *Suillus luteus* (wild) and *Agaricus bisporus* var. *brunnescens* (cultivated), by evaluating their physicochemical characteristics, mineral composition, chromatographically quantified compounds, and in vitro antioxidant, antimicrobial, antiproliferative, anti-inflammatory responses, and *Caenorhabditis elegans* locomotion effects. Furthermore, the study carried out a preliminary investigation into these species as potential sources of functional ingredients.

## 2. Results and Discussion

### 2.1. Qualitative Phytochemical Profile, Physicochemical Characteristics and Mineral Composition

Table 1 presents the qualitative phytochemical profile of the whole mushroom, cap and stipe of *Suillus luteus* and *Agaricus bisporus* var. *brunnescens*. Phenolic compounds and tannins were detected in all the anatomical fractions of both species. Acetogenins were detected exclusively in the whole mushroom, cap and stipe of *A. bisporus* var. *brunnescens*. In contrast, steroids, terpenoids, alkaloids, flavonoids and saponins were not detected in any of the samples under the analytical conditions employed.

Table 2 presents the physicochemical characteristics and mineral composition of the whole mushroom, cap and stipe of both species. The pH varied from 5.6 to 5.8 in *S. luteus* and from 6.4 to 6.6 in *A. bisporus* var. *brunnescens*. A significant difference was observed between the whole mushrooms, whereas no differences were detected when the caps and stipes were compared separately. Titratable acidity remained at approximately 0.1% and did not differ significantly among the samples. Soluble solids ranged from 1.1 to 2.2 °Brix in *S. luteus* and from 1.1 to 1.2 °Brix in *A. bisporus* var. *brunnescens*. Significant differences were found for the whole mushrooms and stipes, but not for the caps. Within *A. bisporus* var. *brunnescens*, the whole mushroom differed significantly from the cap and stipe.

Moisture content ranged from 92.1 to 94.2% in *S. luteus* and from 89.7 to 92.2% in *A. bisporus* var. *brunnescens*. The whole mushroom and cap of *S. luteus* had significantly higher moisture contents than the corresponding fractions of *A. bisporus* var. *brunnescens*, whereas the stipes did not differ significantly between species. Within *A. bisporus* var. *brunnescens*, the stipe had a significantly higher moisture content than the whole mushroom and cap. Ash content was significantly higher in *A. bisporus* var. *brunnescens* than in *S. luteus* for all the anatomical fractions. Within *S. luteus*, the stipe had a lower ash content than the whole mushroom and cap, whereas no differences among fractions were observed in *A. bisporus* var. *brunnescens*.

Potassium was the predominant mineral in both species. *A. bisporus* var. *brunnescens* contained significantly more potassium than *S. luteus* in the whole mushroom, cap and stipe. The highest concentration was recorded in the cap of *A. bisporus* var. *brunnescens* (4899.9 mg/100 g DW), while the stipe exhibited the highest value within *S. luteus* (3839.4 mg/100 g DW). Sodium was also significantly higher in all fractions of *A. bisporus* var. *brunnescens*. Calcium differed significantly between species in the whole mushrooms and caps, but not between the stipes. No significant differences among anatomical fractions were found within either species. Iron ranged from 22.9 to 23.1 mg/100 g DW and did not differ significantly between species or anatomical fractions.

### 2.2. Bioactive Compounds Concentrations in the Edible Mushrooms

Table 3 presents the concentrations of vitamin C, organic acids, total anthocyanins and individual phenolic compounds determined in the whole mushroom, cap and stipe of *Suillus luteus* and *Agaricus bisporus* var. *brunnescens*. Significant differences were identified according to species and anatomical fraction.

Vitamin C concentrations differed significantly between species in all the fractions analysed. The whole mushroom and cap of *A. bisporus* var. *brunnescens* contained significantly higher concentrations than the corresponding fractions of *S. luteus*. The highest value was recorded in the cap of *A. bisporus* var. *brunnescens* (50.2 mg/100 g DW). Conversely, the stipe of *S. luteus* contained significantly more vitamin C than the stipe of *A. bisporus* var. *brunnescens*. Within *S. luteus*, the stipe had a higher concentration than the whole mushroom and cap, whereas the three fractions of *A. bisporus* var. *brunnescens* differed significantly from one another.

Citric, malic and tartaric acids were quantified in both species. Total organic acid content was significantly higher in *S. luteus* than in *A. bisporus* var. *brunnescens* in the whole mushroom, cap and stipe. The highest total concentration was found in the stipe of *S. luteus* (51,974.6 mg/100 g DW), followed by its whole mushroom and cap. Malic acid was the predominant organic acid in *S. luteus* and was significantly more abundant than in the equivalent fractions of *A. bisporus* var. *brunnescens*. In contrast, citric and tartaric acids occurred at significantly higher concentrations in *A. bisporus* var. *brunnescens*.

The total anthocyanin response was significantly higher in *S. luteus* than in *A. bisporus* var. *brunnescens* for all the anatomical fractions. In *S. luteus*, the cap and stipe exhibited similar concentrations and were significantly higher than the whole mushroom. In *A. bisporus* var. *brunnescens*, significant differences were observed among the three fractions, with the cap presenting the highest concentration.

The phenolic profile comprised gallic, 4-hydroxybenzoic, *m*-coumaric and syringic acids. Gallic and *m*-coumaric acids were significantly more abundant in *S. luteus* than in *A. bisporus* var. *brunnescens* across all fractions. The stipe of *S. luteus* contained the highest gallic acid concentration, while *m*-coumaric acid was the predominant quantified phenolic compound in this species.

Conversely, 4-hydroxybenzoic and syringic acids were significantly higher in *A. bisporus* var. *brunnescens*. The highest concentration of 4-hydroxybenzoic acid was found in its cap. Total quantified phenolic compounds were significantly higher in *A. bisporus* var. *brunnescens* than in *S. luteus* for all comparable fractions. The highest total concentration was recorded in the cap of *A. bisporus* var. *brunnescens* (2152.8 mg/100 g DW), although the interspecific difference was less pronounced between the stipes.

### 2.3. In Vitro Antioxidant Activity

Table 4 presents the in vitro antioxidant capacity of the whole mushroom, cap and stipe of *Suillus luteus* and *Agaricus bisporus* var. *brunnescens*, determined using the DPPH and ABTS assays.

The DPPH antioxidant capacity was significantly higher in *S. luteus* than in *A. bisporus* var. *brunnescens* for the whole mushroom, cap and stipe. No significant differences were detected among the fractions of *S. luteus*, which ranged from 3.2 to 3.3 mmol TE/100 g DW. In *A. bisporus* var. *brunnescens*, the whole mushroom and cap did not differ significantly, whereas the stipe exhibited a significantly lower value. Comparison of equivalent fractions showed that the DPPH response of *S. luteus* was approximately 1.1–1.4-fold higher than that of *A. bisporus* var. *brunnescens*.

The ABTS antioxidant capacity was also significantly higher in *S. luteus* than in *A. bisporus* var. *brunnescens* for all three fractions. In *S. luteus*, the whole mushroom presented the highest value (5.9 mmol TE/100 g DW), followed by the cap and stipe, with significant differences among the three fractions. In *A. bisporus* var. *brunnescens*, the cap exhibited the highest ABTS value, followed by the whole mushroom and stipe; all three fractions differed significantly. The ABTS response of *S. luteus* was approximately 1.1–1.4-fold higher than that of the corresponding fractions of *A. bisporus* var. *brunnescens*.

### 2.4. Antimicrobial Activity

Table 5 presents the minimum inhibitory concentrations (MICs) of the hydroethanolic extracts of *Suillus luteus* and *Agaricus bisporus* var. *brunnescens* against eight bacterial and two yeast reference strains. Under the assay conditions, *A. bisporus* showed the broadest growth-inhibitory spectrum, with measurable MICs against all eight bacterial strains. The lowest MIC was recorded against *Staphylococcus aureus* ATCC 6538P (3.9 mg/mL), followed by *Staphylococcus epidermidis* ATCC 12228 (15.6 mg/mL).

The MIC of the *A. bisporus* var. *brunnescens* extract against *S. aureus* was approximately 16-fold lower than that of the *S. luteus* extract. Similarly, its MIC against *S. mutans* was approximately two-fold lower. Conversely, *S. luteus* showed a lower MIC against *E. coli*.

### 2.5. In Vitro Antiproliferative Activity

Table 6 presents the half-maximal inhibitory concentrations (IC_50_) of the mushroom extracts against four tumour cell lines and NIH3T3 non-tumour fibroblasts, together with the corresponding selectivity indices. *S. luteus* exhibited the lowest IC_50_ values across all tumour cell lines, ranging from 0.08 to 0.21 mg/mL, indicating a stronger inhibition of tumour-cell proliferation than *A. bisporus* var. *brunnescens*, for which IC_50_ values ranged from 5.76 to 15.70 mg/mL. In both species, IC_50_ values were relatively consistent across most tumour cell lines, suggesting broad but species-dependent antiproliferative responses; however, HepG2 cells showed lower sensitivity to *A. bisporus* var. *brunnescens*. Among the tested cancer cell lines, THJ29T thyroid carcinoma cells were the most sensitive to *S. luteus*, while HCT116 colorectal cells were the most sensitive to *A. bisporus* var. *brunnescens*. However, the stronger antiproliferative effect of *S. luteus*, which is consistent with its stronger antioxidant profile, was not accompanied by clear tumour-cell selectivity. Its response in NIH3T3 non-tumour fibroblasts was comparable to that observed in several tumour cell lines, resulting in therapeutic index (TI) values below 1 for HeLa, HCT116, and HepG2, and only slightly above 1 for THJ29T. This limits its direct translational relevance despite its strong antiproliferative activity. In contrast, *A. bisporus* var. *brunnescens* displayed a weaker antiproliferative effect but a slightly more favourable selectivity pattern, while exhibiting lower antioxidant activity compared with *S. luteus*. TI values remained close to 1 for HeLa, HCT116, and THJ29T, indicating only modest tumour-cell preference in these models. This observation suggests that tumour-cell selectivity may not depend solely on the overall antioxidant capacity of the extracts. Nevertheless, HepG2 cells represented an exception, showing both the lowest sensitivity and a TI below 1, indicating reduced sensitivity and limited selectivity in the hepatic carcinoma model.

### 2.6. Anti-Inflammatory Activity

**Figure 1:**
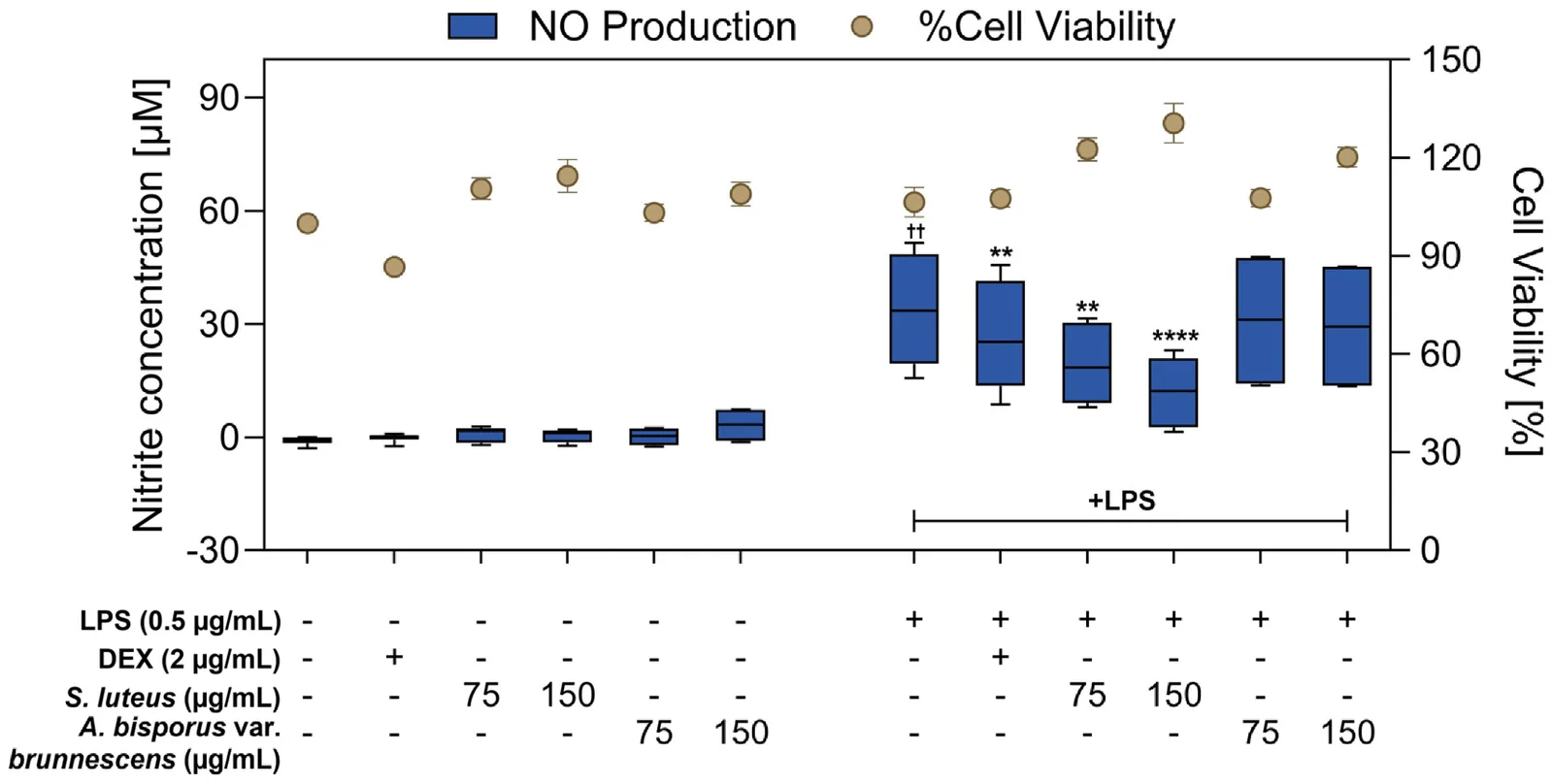
Effects of Suillus luteus and Agaricus bisporus var. brunnescens extracts on LPS-induced nitric oxide-related production in RAW 264.7 macrophages. Blue boxplots represent nitrite accumulation, and gold symbols represent cell viability determined by crystal violet staining. DEX: dexamethasone. Data represent at least four independent biological replicates. ††, *p* < 0.01 versus Control −LPS; **, adjusted *p* < 0.01 versus Control + LPS; ****, adjusted *p* < 0.0001 versus Control + LPS. Statistical symbols refer exclusively to nitrite concentrations. DEX, dexamethasone; LPS, lipopolysaccharide.

LPS stimulation markedly increased nitrite accumulation in RAW 264.7 macrophages, reaching a mean concentration of 34.01 µM (Figure 1). Dexamethasone significantly attenuated this response, reducing nitrite accumulation by 40.5% to 20.25 µM and confirming assay responsiveness. *S. luteus* significantly reduced LPS-induced nitrite accumulation by 43.3% and 64.6%, reaching mean concentrations of 19.30 and 12.05 µM at 75 and 150 µg/mL, respectively. In contrast, the mean concentrations observed with *A. bisporus* var. *brunnescens* remained close to the LPS-stimulated control, reaching 30.98 and 29.34 µM, and neither response was statistically significant. Nitrite levels remained close to baseline in the absence of LPS. In parallel, crystal violet staining showed no reduction in cell viability or adherent cell biomass. Together with the previous MTT assay, these results indicate that the lower nitrite accumulation observed in *S. luteus* was not due to cytotoxicity or reduced cell number.

### 2.7. Nematode Locomotion-Disrupting Activity

**Figure 2:**
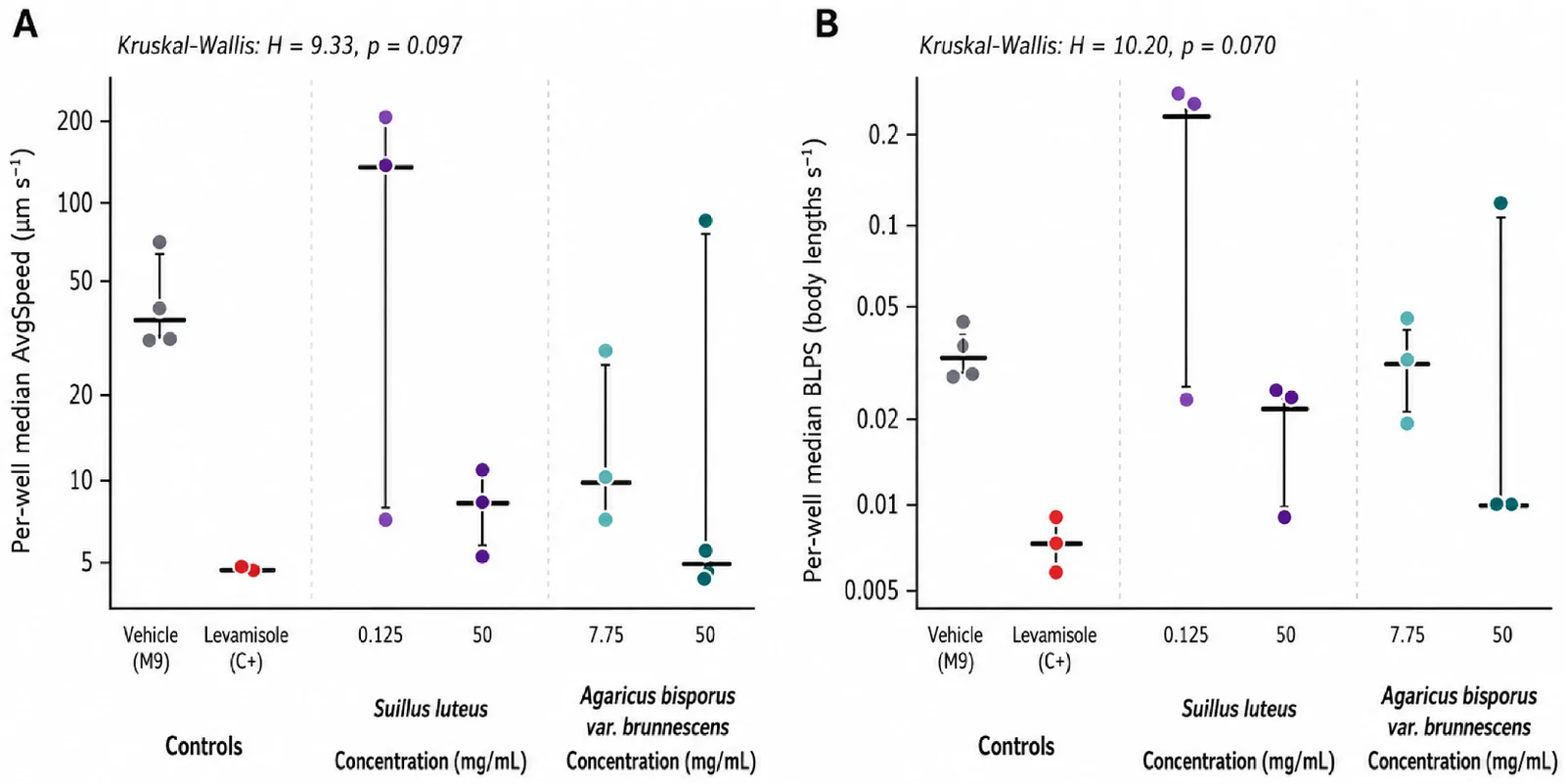
Nematode locomotion-disrupting activity of *Suillus luteus* and *Agaricus bisporus* var. *brunnescens* extracts in *Caenorhabditis elegans*. (**A**) Locomotion speed (AvgSpeed, µm/s) and (**B**) normalised locomotion speed (BLPS, body lengths/s) following exposure to fungal extracts. *S. luteus* was evaluated at 0.125 and 50 mg/mL, whereas *A. bisporus* var. *brunnescens* was evaluated at 7.75 and 50 mg/mL. M9 buffer was used as the negative control (C−), and 2% levamisole was used as the positive locomotion-inhibition control (C+). Three independent videos obtained from independent wells were analysed per experimental condition (n = 3). Each circle represents the median of all individual tracks detected in a single independent video. The thick horizontal line represents the median of the three video-level observations, and the vertical whiskers represent the minimum–maximum range. Y-axes are displayed on a logarithmic scale. Global differences among experimental conditions were evaluated using the Kruskal–Wallis test; the corresponding H and *p* values are displayed above each panel. Statistical significance was defined as *p* < 0.05.

The locomotor response of *Caenorhabditis elegans* to the fungal extracts was evaluated using AvgSpeed and BLPS as the principal locomotion parameters (Figure 2). Under M9 control conditions, nematodes exhibited a median AvgSpeed of 38.127 µm/s and a median BLPS of 0.038 body lengths/s. As expected, levamisole markedly impaired locomotion, reducing median AvgSpeed to 4.088 µm/s and BLPS to 0.007 body lengths/s, corresponding to reductions of approximately 89.3% and 81.6%, respectively, relative to the M9 control.

For *S. luteus*, exposure to 50 mg/mL produced a marked reduction in both locomotion parameters. Median AvgSpeed decreased to 7.997 µm/s, representing a 79.0% reduction relative to the M9 control, while median BLPS decreased to 0.019 body lengths/s, corresponding to a 50.0% reduction. In contrast, exposure to 0.125 mg/mL did not produce a locomotion-reducing response. At this concentration, median AvgSpeed and BLPS were 145.661 µm/s and 0.223 body lengths/s, respectively, both exceeding the corresponding M9-control medians.

For *A. bisporus* var. *brunnescens*, exposure to 7.75 mg/mL reduced median AvgSpeed to 10.190 µm/s, corresponding to a 73.3% reduction relative to M9. However, the median BLPS remained close to the control value of 0.035 body lengths/s, representing a reduction of approximately 7.9%. At 50 mg/mL, median AvgSpeed and BLPS decreased to 4.298 µm/s and 0.010 body lengths/s, corresponding to reductions of approximately 88.7% and 73.7%, respectively, relative to the M9 control.

**Figure 3:**
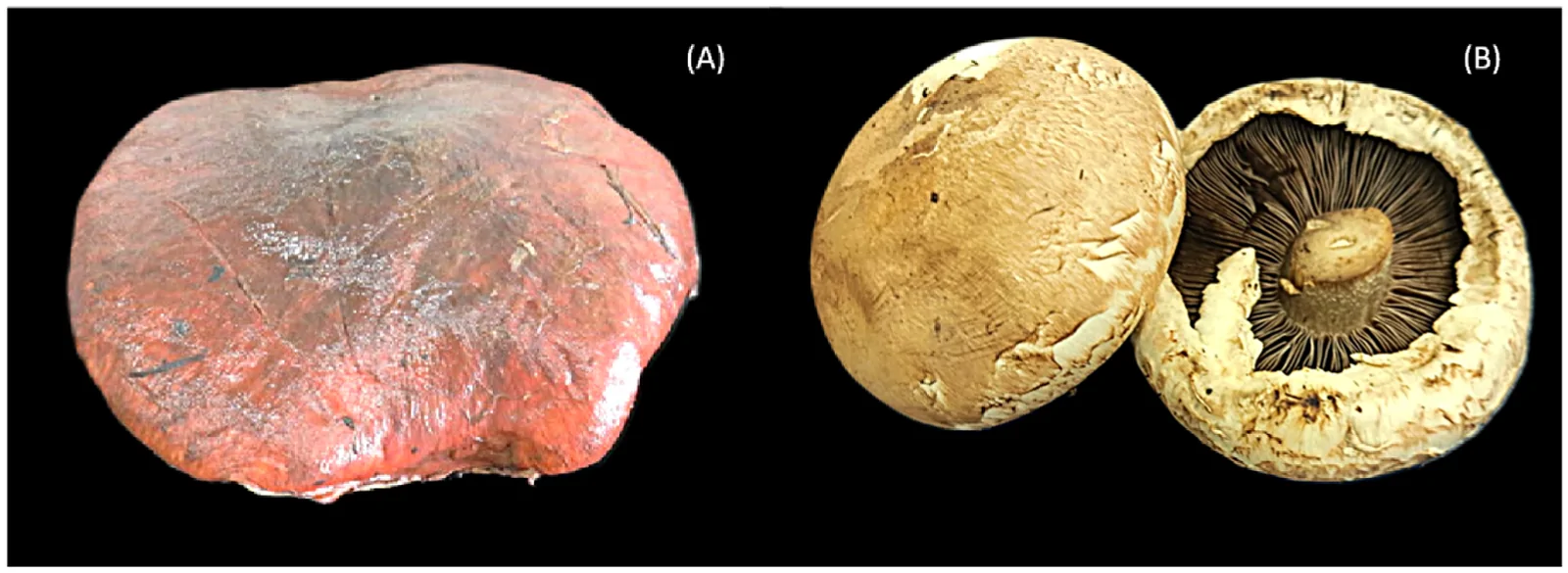
Photograph of edible fungi. Note: (**A**), *Suillus luteus* (L.) Roussel (*Pine mushroom*); (**B**), *Agaricus bisporus* var. *brunnescens* (Portobello coffee).

Despite the magnitude of several of these descriptive reductions, the global comparisons did not reach the conventional threshold for statistical significance (*p* < 0.05). The complete distribution of the three independent video-level observations, including their minimum–maximum ranges and the corresponding Kruskal–Wallis statistics, is presented in Figure 3. Accordingly, the observed differences should be interpreted as descriptive locomotor responses rather than statistically confirmed treatment effects.

## 3. Discussion

### 3.1. Qualitative Phytochemical Profile, Physicochemical Characteristics and Mineral Composition

The detection of phenolic compounds and tannins in all the analysed fractions indicates that these chemical groups are distributed throughout the fruiting bodies of both species. Nevertheless, qualitative colour reactions provide preliminary evidence of broad metabolite classes and cannot establish the identity or concentration of individual compounds. Their response may be affected by solvent polarity, extraction efficiency, analyte concentration and reaction specificity. Consequently, a negative response indicates that the corresponding chemical group was not detected under the conditions employed and should not be interpreted as definitive evidence of its absence. Variations in the qualitative detection of phenols, tannins, flavonoids, saponins and terpenoid compounds have similarly been associated with fungal species and extraction conditions [11].

This limitation is particularly relevant to *A. bisporus*, for which chromatographic and mass spectrometric analyses have identified phenolic acids, amino acids, fatty acids, steroids, and peptides that may not be detected by conventional colourimetric screening [9].

The slightly acidic pH and low titratable acidity observed in both species are consistent with the physicochemical characteristics of fresh mushrooms. Moisture was high in every fraction, confirming the predominance of water in fresh fruiting bodies. The values obtained for *A. bisporus* var. *brunnescens* were within the 85–95% interval reported for fresh *Agaricus* fruiting bodies [12]. The moisture content of *S. luteus* largely overlapped the 84.5–93.9% range previously reported for this species, although both the whole mushroom and the cap were marginally above the upper limit. Differences among studies may be associated with developmental stage, environmental conditions and postharvest handling, although the contribution of these factors cannot be established from the present experimental design [13].

The higher ash content of *A. bisporus* var. *brunnescens* indicates a greater total inorganic residue than that of *S. luteus*. However, ash represents the combined mineral residue and cannot by itself identify which elements account for the interspecific difference. Mineral accumulation in mushrooms is influenced by species, strain, substrate composition, cultivation system and environmental availability [12,14].

Potassium predominated in every anatomical fraction, in agreement with its recognition as one of the major mineral elements in edible mushrooms. Its greater concentration in *A. bisporus* var. *brunnescens* demonstrates a species-dependent mineral profile, while the differences among caps, stipes and whole fruiting bodies show that mineral accumulation may also vary according to anatomical fraction. The significantly higher sodium concentrations found in *A. bisporus* var. *brunnescens* followed the same interspecific pattern, although sodium remained markedly less abundant than potassium [15,16].

### 3.2. Bioactive Compound Concentrations in the Edible Mushrooms

The distribution of vitamin C differed between the two mushrooms, demonstrating that the anatomical fraction containing the highest concentration was species-dependent. The cap was the principal accumulation fraction in *A. bisporus* var. *brunnescens*, whereas the stipe contained the highest concentration in *S. luteus*. Vitamin concentrations in edible mushrooms can be affected by genetic characteristics, substrate composition, developmental stage, environmental conditions, processing and storage [5].

The predominance of malic acid accounted for the higher total organic acid concentration in *S. luteus*, particularly in the stipe. In contrast, *A. bisporus* var. *brunnescens* was characterised by greater concentrations of citric and tartaric acids. This demonstrates that the difference between species involved not only the total concentration but also the relative distribution of the individual acids. These compounds participate in primary metabolism and can influence acidity, flavour and the chemical stability of mushroom tissues. Citric and malic acids have previously been identified by chromatography in A. bisporus, although their concentrations vary with the biological material and analytical procedure [17]. Variability in the organic acid profiles of wild edible mushrooms has also been associated with taxonomic identity, growing conditions and geographical origin [18].

Fungal fruiting bodies contain chemically diverse pigments, some of which may exhibit overlapping absorption within the spectral region used for anthocyanin determination. Therefore, these results demonstrate a species and fraction [19].

*S. luteus* was characterised by greater concentrations of gallic and *m*-coumaric acids, whereas *A. bisporus* var. *brunnescens* contained more 4-hydroxybenzoic and syringic acids. The identification of phenolic acids in *A. bisporus* is consistent with instrumental studies employing high-resolution chromatographic and mass spectrometric techniques. Nevertheless, direct comparison of absolute concentrations among studies remains limited by differences in mushroom variety, extraction solvent, sample-to-solvent ratio and analytical conditions [9].

### 3.3. In Vitro Antioxidant Activity

The values obtained with the ABTS assay were higher than those from the DPPH assay in all samples. However, this difference should not be interpreted as indicating greater absolute antioxidant activity, since the assays employ different radicals, reaction times, and chemical conditions. ABTS may respond to a broader range of hydrophilic and lipophilic substances, whereas the DPPH response depends particularly on the solubility of the extracts in the reaction medium, their accessibility to the radical, and the kinetics of electron or hydrogen atom transfer [20].

The profile of *S. luteus* was characterised by higher concentrations of gallic acid and m-coumaric acid, whereas *A. bisporus* var. *brunnescens* exhibited higher concentrations of 4-hydroxybenzoic acid and syringic acid. These qualitative differences could contribute to the observed behaviour, but the results do not permit attributing the antioxidant capacity to a specific compound because the analytes were not evaluated individually. The antioxidant capacity of fungi depends on both the concentration and the specific profile of the extract, as well as their possible interactions within the matrix [14].

The antioxidant capacity of *S. luteus* is consistent with recent studies reporting the presence of polyphenols and a response to the DPPH radical in samples of this species. However, the absolute values are not directly comparable due to differences in sample origin, drying method, extraction solvent, and reporting format [13]. The antioxidant activity of *A. bisporus* may also vary depending on the strain, substrate, developmental stage, extraction procedure, and chemical method used [14,15].

### 3.4. Antimicrobial Activity

The extract of *A. bisporus* var. *brunnescens* exhibited a broader antibacterial spectrum than that of *S. luteus*, with inhibitory responses against all the Gram-positive and Gram-negative strains evaluated. Nevertheless, its effectiveness varied markedly among microorganisms. The lowest MICs were observed against the Gram-positive species *S. aureus* and *S. epidermidis*, whereas substantially higher concentrations were required to inhibit the Gram-negative species *Pseudomonas* and *E. coli*. This pattern suggests greater activity against some Gram-positive bacteria but does not establish a response determined exclusively by Gram classification, as the extract also inhibited *E. tarda* and *P. putida* at intermediate concentrations.

The particularly high activity of *A. bisporus* var. *brunnescens* against the two *Staphylococcus* species is qualitatively consistent with recent studies demonstrating the antibacterial activity of ethanolic and acetonic extracts from white and brown *A. bisporus* species, including against *S. aureus* strains. However, direct comparison of MIC values should be approached with caution, as there are differences in the extraction solvent, extract composition, bacterial strains, and experimental procedures [9].

The differential response among microorganisms is also consistent with the variability described for basidiomycete extracts. The antimicrobial activity of these materials depends on the fungal species, the origin and growth conditions of the fruiting body, the extraction procedure, and the sensitivity of the strain used. Furthermore, crude extracts contain complex mixtures of metabolites; therefore, the results obtained do not allow the inhibition to be attributed to a specific compound [21,22].

No antifungal activity was detected for either extract against *Candida albicans* ATCC 10231 or *Candida tropicalis* ATCC 13803 within the tested concentration range. Because only two reference strains were evaluated, this result should be interpreted as specific to the strains and assay conditions used, rather than as evidence that the mushroom species lack antifungal potential. Susceptibility may vary among *Candida* species and strains and may also depend on extract composition, solvent polarity, fractionation, and concentration [21,22]. Broader screening with additional yeast strains, together with extract fractionation, will therefore be needed to determine whether these mushrooms contain antifungal constituents.

The extraction condition used in this study was previously selected to maximise phenolics recovery. Nevertheless, this criterion does not necessarily represent the optimum condition for extracting all bioactive constituents present in the mushrooms. A major limitation is that only MIC values were determined. The MIC identifies the lowest concentration of the extract that produces total growth inhibition under the test conditions, but does not demonstrate microbial eradication. As no MBC or eradication time analyses were carried out, the present results do not allow a distinction to be made between bacteriostatic and bactericidal effects.

### 3.5. Antiproliferative Activity and Tumour-Cell Selectivity

The lower IC_50_ values obtained with *S. luteus* indicate a stronger in vitro antiproliferative response than that produced by *Agaricus bisporus* var. *brunnescens*. Nevertheless, potency alone does not establish tumour-cell selectivity. The response of NIH3T3 non-tumour fibroblasts to *S. luteus* was comparable to that observed in several tumour cell lines, resulting in selectivity indices below 1 for HeLa, HCT116 and HepG2 cells. Only THJ29T cells showed limited preferential sensitivity, with an index slightly above 1. Thus, despite its strong growth-inhibitory effect, the crude extract did not demonstrate consistent selectivity towards tumour cells.

In contrast, *A. bisporus* var. *brunnescens* exhibited higher IC_50_ values but showed little tumour-cell selectivity in HeLa, HCT116 and THJ29T cells, as indicated by TI values close to unity. HepG2 cells showed the lowest sensitivity to this extract and a selectivity index below 1. Accordingly, neither mushroom extract demonstrated strong and consistent selectivity across the four tumour-cell models.

The antiproliferative responses may be associated with the chemically diverse constituents of the crude extracts. Edible mushrooms contain phenolic compounds, polysaccharides, terpenoids, sterols, proteins and other metabolites capable of affecting oxidative signalling, cell-cycle progression and apoptosis-related pathways. Nevertheless, the relative contribution of these compound classes depends on the fungal species, extraction procedure, concentration and cellular model [23].

The phenolic profiles differed markedly between the two mushroom species. *S. luteus* contained higher concentrations of gallic and *m*-coumaric acids, whereas *A. bisporus* var. *brunnescens* contained more 4-hydroxybenzoic and syringic acids. Phenolic acids have been reported to modulate oxidative stress, proliferation, cell-cycle regulation and apoptosis-related processes. However, the present results do not establish that the quantified phenolic acids were directly responsible for the observed antiproliferative effects because the individual compounds were not evaluated separately [24,25]. Furthermore, the broad antitumour potential of edible fungi has been extensively documented, highlighting their role as sources of therapeutically relevant bioactive compounds [23]. These findings are consistent with previous studies reporting cell-line- and extract-dependent antiproliferative effects for *S. luteus* and other edible mushroom extracts in colon cancer, HeLa, HepG2, and other tumour-cell models [26,27,28,29]. However, the selectivity and magnitude of response depend on species, extraction method, exposure time, assay endpoint, and tumour-cell model. Nevertheless, the specific mechanisms underlying the effects observed in the present study remain undetermined, as apoptosis and cell-cycle progression were not directly evaluated.

Taken together, these data show distinct antiproliferative and selectivity profiles between the two species. *S. luteus* showed stronger inhibition of cell proliferation, although neither crude extract demonstrated consistent tumour-cell selectivity. Nevertheless, their antiproliferative activity provides a rationale for bioactivity-guided fractionation. Evaluating the resulting fractions and isolated constituents in both tumour and non-tumour cells could determine whether the observed activity can be retained with improved selectivity.

### 3.6. Anti-Inflammatory Activity

The significant and concentration-dependent reduction in nitrite accumulation produced by *S. luteus* demonstrates that its hydroethanolic extract attenuated the LPS-induced response in RAW 264.7 macrophages. At 150 µg/mL, the reduction exceeded that produced by dexamethasone under the experimental conditions. The absence of a reduction in cell viability or adherent biomass supports the interpretation that the lower nitrite concentrations observed following treatment with *S. luteus* were not solely attributable to cytotoxicity or cell loss.

The different responses of the two mushroom extracts may be related to their phenolic profiles. Chemical analysis showed that *S. luteus* contained considerably more *m*-coumaric acid and higher concentrations of gallic acid than *A. bisporus* var. *brunnescens* (Table 3). Gallic acid has been shown to reduce LPS-induced nitrite accumulation in RAW 264.7 macrophages [30]. Although *m*-coumaric acid has not been evaluated directly in this model, its positional isomer, *p*-coumaric acid, reduced nitrite production in the same assay, making a similar contribution plausible due to their shared hydroxycinnamic acid structure [31].

*A. bisporus* var. *brunnescens* contained higher concentrations of 4-hydroxybenzoic acid and vitamin C than *S. luteus*, but this did not translate into significant inhibition. Both compounds have previously reduced LPS-induced NO-related responses in RAW 264.7 macrophages [32]. Because each compound represented only a fraction of the 150 µg/mL extract, its concentration in the cellular treatment may have remained below the ranges reported to be active. To the best of our knowledge, no previous study has evaluated *S. luteus* in this cellular model. Nevertheless, a methanolic fruiting-body extract of *Suillus bellinii*, from the same genus and family, inhibited nitrite production with an EC_50_ of 90 µg/mL [29].

For *A. bisporus*, an EC_50_ of 190 µg/mL was reported for one sample, whereas the Portobello sample remained inactive within the evaluated concentration range [31]. In LPS-stimulated RAW 264.7 macrophages, a methanolic extract tested at 0.5 mg/mL reduced nitrite accumulation and downregulated the expression of iNOS, IL-1β, and IL-6 [33]. Additionally, raw preparations of white button and honey brown *A. bisporus* inhibited NO production in LPS- and IFN-γ-activated RAW 264.7 macrophages [34]. Together, these variable findings suggest that the activity of *A. bisporus* depends on mushroom type, extract preparation, and the concentration tested. Overall, the significant inhibition produced by *S. luteus*, together with its phenolic profile and the activity reported for related compounds and *S. bellinii*, supports the anti-inflammatory potential of this species in the present in vitro model. Nevertheless, nitrite accumulation is an indirect indicator of nitric oxide-related production and represents only one component of the inflammatory response. Therefore, the results demonstrate inhibition of LPS-induced nitrite accumulation but do not, by themselves, establish a comprehensive anti-inflammatory mechanism. Future studies focused on this species should examine whether the observed reduction in nitrite accumulation is accompanied by changes in IL-1β and TNF-α levels and in iNOS and COX-2 expression.

### 3.7. Nematode Locomotion-Disrupting Activity

Both mushroom extracts produced substantial descriptive reductions in locomotor parameters at 50 mg/mL. At this concentration, *S. luteus* reduced both AvgSpeed and BLPS, while *A. bisporus* var. *brunnescens* produced reductions approaching those observed with levamisole. Nevertheless, neither AvgSpeed nor BLPS showed a statistically significant global difference among the experimental conditions. The two locomotor parameters did not respond uniformly. At 7.75 mg/mL, *A. bisporus* var. *brunnescens* produced a marked descriptive reduction in AvgSpeed, while BLPS remained close to the vehicle-control median. AvgSpeed is expressed as absolute distance travelled per unit of time, whereas BLPS adjusts speed according to the body length of the tracked organism. Previous studies support the ability of edible basidiomycetes to affect nematode survival and locomotor behaviour. Wille et al. [35] evaluated aqueous extracts of several basidiomycetes against *Meloidogyne incognita* and reported marked nematicidal activity for extracts obtained from *Suillus* sp., supporting the presence of nematode-active constituents within this fungal genus. Similarly, da Silva et al. [36] demonstrated that edible *Pleurotus* species impaired nematode motility in *Panagrellus* sp. and associated this activity with fungal proteases, providing additional evidence that edible mushroom-derived constituents may directly interfere with nematode locomotor function.

Interestingly, the response to *A. bisporus* appears to depend strongly on the experimental context. Caetano et al. reported that *A. bisporus* at 100 mg/mL improved motility in lead-exposed *C. elegans*, indicating a protective effect under chemically induced stress [37]. This contrasting observation suggests that the effects of mushroom extracts on nematode locomotion may vary with fungal species, extract composition and concentration, exposure conditions, and the nematode’s physiological status. Although differences among conditions did not reach statistical significance for AvgSpeed (Kruskal–Wallis, H = 9.33, *p* = 0.097) or BLPS (H = 10.20, *p* = 0.070), several treatments produced marked descriptive changes relative to the M9 vehicle control. These responses were accompanied by substantial between-well variability, and only three independent wells were analysed per condition. The assay therefore had limited power to distinguish treatment effects from experimental variation. Accordingly, the non-significant results should be interpreted cautiously and do not exclude a locomotor effect. These findings are exploratory and require confirmation with a larger number of independent experimental units.

## 4. Materials and Methods

### 4.1. Physicochemical Analyses

Two edible mushroom taxa were evaluated: the wild species *Suillus luteus* and the cultivated mushroom *Agaricus bisporus* var. *brunnescens* (Figure 3). *S. luteus* fruiting bodies were collected from a pine forest located in Ayora, Cayambe Canton, Pichincha Province, Ecuador. Ayora is situated in the Ecuadorian Andes at approximately 2860–2900 m above sea level and is characterised by an equatorial cold-humid to semi-humid climate, with a mean annual temperature of approximately 15 °C. *Agaricus bisporus* var. *brunnesens* was acquired in Tumbaco, Pichincha Province, Ecuador. Fruiting bodies were selected when they had reached complete morphological development. For each mushroom taxon, three separate lots of 50 fruiting bodies were obtained. Each lot was divided into two portions: one was used for physicochemical analyses, whereas the other was frozen and subsequently freeze-dried using a Christ Alpha 1-4 LDplus freeze-dryer (Martin Christ Gefriertrocknungsanlagen GmbH, Osterode am Harz, Germany). Within each portion, the fruiting bodies were further allocated to independent sample groups. One group was analysed as intact whole fruiting bodies, comprising both the cap and the stipe. A separate group was dissected, and its caps and stipes were processed independently. Consequently, the whole-fruiting-body samples were not obtained by recombining the separately analysed caps and stipes. Each sample group was homogenised and analysed independently.

Commercial quality assessment included pH measurement, carried out with a digital SevenMultiS47 potentiometer (Mettler Toledo, Columbus, OH, USA), whilst soluble solids (°Brix) were determined with a manual refractometer Hitech RHB-32 (G-Won Hitech Co., Ltd., Seoul, Republic of Korea). Total titratable acidity (TA) was determined by titration with 0.1 M NaOH and expressed as a percentage. Moisture content was determined by oven drying at 105 °C to constant weight using a Memmert Be20 (Memmert GmbH+Co.KG, Barcelona, Spain), and ash content was determined by incineration in a muffle furnace (Thermo Fisher Scientific, Waltham, MA, USA) at 550 °C. All analyses were carried out in triplicate.

The preliminary phytochemical characterisation of the samples under study was carried out using colourimetric assays designed for the qualitative detection of alkaloids, tannins, saponins, acetogenins, steroids, terpenoids, phenolic compounds and flavonoids in accordance with the methodology outlined by León-Fernández et al. [38]. To this end, 20 mg of freeze-dried mushroom material was suspended in 1 mL of deionised water, then homogenised and sonicated for 3 min in an FS60D ultrasonic bath (Intebiolab Inc., Orlando, FL, USA). Subsequently, the samples were centrifuged at 14,000 rpm and 4 °C for 5 min using an Eppendorf 5430 centrifuge (Eppendorf AG, Hamburg, Germany). The supernatant obtained was recovered, whilst the solid residue was subjected to two additional extraction steps with 500 µL of deionised water each. Finally, the combined supernatants constituted the extract used for the phytochemical determinations.

The determination of calcium, iron, potassium, and sodium was carried out using atomic absorption spectrophotometry with a Varian Spectra A-55 (Varian Inc., Palo Alto, CA, USA) in accordance with the methodology outlined by Coyago-Cruz et al. [39]. The samples were subjected to microwave-assisted digestion in a Speedwave Xpert system (Berghof Products + Instruments GmbH, Eningen unter Achalm, Germany), equipped with Teflon vessels. Briefly, 40 mg of the freeze-dried sample was treated with 5 mL of 65% nitric acid and digested under conditions specified by the manufacturer. Once digestion was complete, the extracts were brought to a final volume of 25 mL with deionised water. The wavelengths used for quantification were 422.7 nm for calcium, 372.0 nm for iron, 404.4 nm for potassium, and 589.6 nm for sodium. All analyses were performed in triplicate, and the results were expressed as mg/100 g dry weight.

### 4.2. Analysis of Bioactive Compounds by Liquid Chromatography

The bioactive constituents of the samples under study were characterised by quantifying vitamin C, organic acids, carotenoids, chlorophyll pigments, and phenolic compounds [40]. Lyophilised samples (20 mg) were subjected to microextraction procedures, including homogenisation and ultrasound-assisted extraction, in an FS60 ultrasonic bath (Fisher Scientific Inc., Waltham, MA, USA). Following extraction, samples were centrifuged at 14,000 rpm for 5 min at 4 °C using an Eppendorf 5430 centrifuge (Eppendorf AG, Hamburg, Germany). The recovered supernatants were filtered through 0.45 µm PVDF membranes before chromatographic analysis.

Compound separation and quantification were performed using an RRLC 1200 systipe coupled to a DAD-UV-Vis detector (Agilent Technologies, Santa Clara, CA, USA). Analytical conditions, including chromatographic columns, detection wavelengths, injection volumes, and mobile phases, were optimised for the target compound group while maintaining a constant flow rate of 1 mL/min. Identification was based on retention times and spectral characteristics, using Lab ChemStation software (v. 2.15.26), compared with authentic standards. Quantification was achieved through external calibration curves prepared from analytical-grade standards (1 mg/mL). Sample extraction was conducted in triplicate, and chromatographic measurements were performed in duplicate. Results were expressed as mg/100 g dry weight, while total contents corresponded to the sum of individual compounds.

For vitamin C analysis, samples were extracted with metaphosphoric acid (3%) and DL-homocysteine (0.2%), then homogenised, agitated, and diluted with deionised water. After centrifugation and filtration, extracts were analysed at 244 nm using a ZORBAX Eclipse XDB-C18 column (1.8 µm, 4.6 × 50 mm). The mobile phase consisted of monobasic potassium phosphate (1.5%) and n-acetyl-n,n,n-trimethylammonium bromide (1.8%) dissolved in methanol (90:10, *v*/*v*). Quantification was carried out using *L*-(+) ascorbic acid as the reference standard.

Organic acids were extracted using sulphuric acid (0.02 N) containing metaphosphoric acid and *DL*-homocysteine. Chromatographic separation was performed at 210 nm using a YMC-Triart C18 column (3 µm, 4.6 × 150 mm) and an isocratic mobile phase consisting of 0.027% sulphuric acid. Calibration curves of citric acid, malic acid, and tartaric acid were used for quantification.

Carotenoids and chlorophyll-related compounds were extracted with a methanol:acetone:dichloromethane mixture (1:1:2), followed by successive dichloromethane extractions until complete pigment removal. The pooled extracts were concentrated under reduced pressure at 30 °C, reconstituted in ethyl acetate and analysed on a YMC C30 column (3 µm, 4.6 × 150 mm). Detection was performed between 250 and 500 nm using methanol, methyl tert-butyl ether and water as mobile phases. Quantification was based on external standards of individual carotenoids and chlorophyll derivatives.

**Figure 4:**
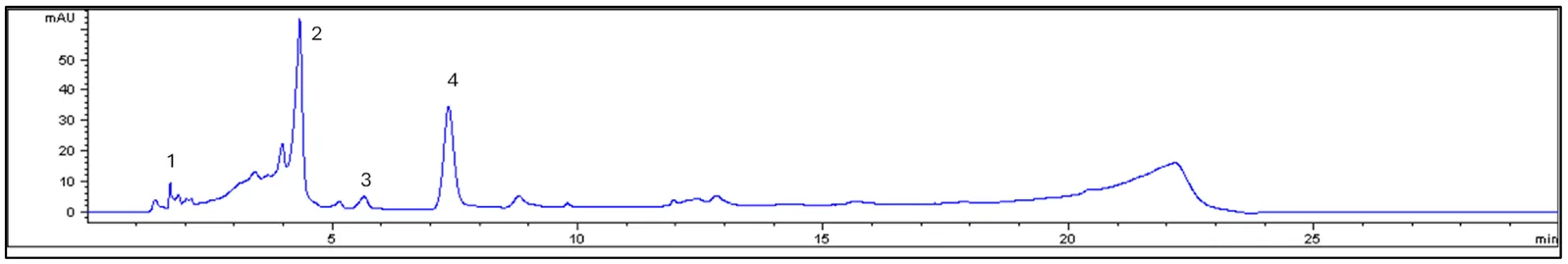
Representative RRLC-DAD chromatogram of the phenolic profile of whole *Agaricus bisporus* var. *brunnescens* at a wavelength of 280 nm. Note: 1, gallic acid; 2, 4-hidroxybenzoic acid; 3, m-coumaric acid; 4, syringic acid.

Phenolic compounds were extracted using acidified methanol (80%, 0.1% HCl), followed by two additional extraction cycles. Separation was achieved on a Zorbax Eclipse Plus C18 column (5 µm, 4.6 × 150 mm) employing a gradient of aqueous formic acid (0.01%) and acetonitrile. Individual phenolics were quantified using calibration curves generated from authentic phenolic acids, flavonoids, and related standards. An representative RRLC-DAD chromatogram of phenolics detected in *Agaricus bisporus* var. *brunnescens* is presented in Figure 4. The target compounds showed distinguishable chromatographic peaks under the analytical conditions employed.

### 4.3. Total Anthocyanin Analyses

Total anthocyanins were determined using the pH differential method [41]. For each fraction of the fruiting body—whole mushroom, cap, and stipe—40 mg of freeze-dried, powdered sample was weighed. Subsequently, 2 mL of 96% ethanol was added; the suspension was homogenised and subjected to ultrasound-assisted extraction for 3 min. The resulting extract was filtered through a PVDF membrane with a pore size of 0.45 µm.

Two 50 µL aliquots were taken from each filtrate and dispensed separately into a 96-well microplate. The first aliquot was combined with 200 µL of 0.025 M potassium chloride buffer, adjusted to pH 1.0, whilst the second was mixed with 200 µL of 0.4 M sodium acetate buffer, adjusted to pH 4.5. The absorbance of both preparations was recorded at 520 and 700 nm using a microplate reader.

Quantification was performed using a cyanidin-3-glucoside calibration curve prepared over the range 0.05–0.20 mg/mL. Three independent extractions were performed for each fraction, and each extract was measured in duplicate. The results were expressed as milligrams equivalent of cyanidin-3-glucoside per 100 g of dry weight—mg EC_3_G/100 g DW.

### 4.4. Antioxidant Activity Analyses

The antioxidant capacity of the extracts under study was evaluated using the ABTS and DPPH radical-scavenging assays [42]. Measurements were performed on a BioTek H1 microplate reader (Agilent Scientific Instruments, Santa Clara, CA, USA). Methanolic extracts were obtained by combining 20 mg of freeze-dried sample with 2 mL of methanol, followed by homogenisation and ultrasound-assisted extraction for 3 min using an FS60 ultrasonic bath (Fisher Scientific Inc., Waltham, MA, USA). The resulting suspensions were centrifuged at 14,000 rpm for 5 min at 4 °C in an Eppendorf 5430 centrifuge (Eppendorf AG, Hamburg, Germany). After filtration through 0.45 µm PVDF membranes, the extracts were used for antioxidant analyses. Three independent extractions were performed per sample, and each extract was analysed in duplicate. Quantification was carried out using a Trolox standard curve (0.2 mg/mL), and the results were expressed as mmol Trolox equivalents (TE)/100 g dry weight.

For the ABTS assay, the radical cation was generated by reacting 7 mM ABTS with 2.45 mM potassium persulfate in the dark for 16 h. Prior to analysis, the solution was diluted with methanol to obtain an absorbance value of 0.70 at 734 nm. Antioxidant activity was determined by mixing 10 µL of extract with 200 µL of the ABTS working solution and incubating the mixture for 15 min before absorbance measurement.

The DPPH assay was conducted using a solution prepared by dissolving 10 mg of DPPH in 50 mL of methanol. The absorbance was adjusted to a range of 0.9–1.1 at 515 nm. Subsequently, 20 µL of extract was mixed with 180 µL of DPPH solution and stored in the dark at room temperature for 40 min before absorbance determination.

### 4.5. Antimicrobial Activity Analyses

Hydroethanolic extracts were prepared from the freeze-dried mushroom samples, as described in the methodology by Coyago et al. [42]. The hydroethanolic solvent was established on the basis of a previous experimental, in which different extraction solvents or water–ethanol proportions were compared using phenolic-compound recovery as the response variable. Among the conditions evaluated, 50% ethanol (*v*/*v*) provided the most favourable recovery of phenolic compounds. Thus, 2 g of each powdered sample was mixed with 25 mL of 50% ethanol and homogenised for 6 min. After extraction, the suspension was centrifuged, and the supernatant was recovered. The solid residue was subjected to two additional extraction cycles under the same conditions. The resulting supernatants were combined, filtered, and concentrated under reduced pressure at a temperature not exceeding 40 °C. The concentrated extracts were freeze-dried and stored in airtight containers protected from light until antimicrobial evaluation. Extraction yield was calculated as the percentage ratio between the mass of the freeze-dried extract and the initial dry mass of the mushroom sample.

#### 4.5.1. Antibacterial Activity

The antibacterial activity of the mushroom extract was evaluated by the broth microdilution method in sterile 96-well microplates, following a procedure adapted from the Clinical and Laboratory Standards Institute (CLSI) [43]. The following strains were used: *Escherichia coli* ATCC 8739, *Pseudomonas aeruginosa* ATCC 9027, *Staphylococcus aureus* ATCC 6538P, *Streptococcus mutans* ATCC 25175, *Pseudomonas fluorescens* ATCC 13525, *Staphylococcus epidermidis* ATCC 12228, *Pseudomonas putida* ATCC 31483, and *Edwardsiella tarda* ATCC 15947.

Stock solutions were prepared by dissolving 300 mg of each lyophilised extract in 1 mL of sterile distilled water. From these solutions, twofold serial dilutions were prepared in sterile 96-well microplates containing 100 µL of brain–heart infusion (BHI) broth. To each well, 20 µL of a bacterial suspension was added, initially standardised to an OD_600_ of 0.2 and subsequently adjusted to an approximate final concentration of CFU/mL. The final reaction volume was 120 µL.

The plates were incubated for 24 h at 37 °C, except for those containing *P. fluorescens*, which were kept at 29 °C. Upon completion of the incubation, 20 µL of 2,3,5-triphenyltetrazolium chloride (TTC) was added to each well. The plates were incubated again for 1–2 h, protected from light. Formazan formation was considered indicative of metabolic activity and, therefore, of microbial growth. The minimum inhibitory concentration (MIC) was defined as the lowest concentration of the extract at which no formation of the coloured product was observed.

Growth, sterility, and solvent controls were included, along with streptomycin as a positive antibacterial control. All treatments were analysed in triplicate. Due to the adaptations used to evaluate crude mushroom extracts, the MICs obtained were interpreted as experimental inhibitory concentrations rather than clinical susceptibility cut-off points.

The assay was designed to determine MIC as an endpoint of growth inhibition. Minimum bactericidal concentration was not determined because aliquots from wells without visible growth were not quantitatively subcultured onto extract-free agar to assess surviving bacterial populations. Consequently, the present results do not distinguish between bacteriostatic and bactericidal effects.

#### 4.5.2. Antifungal Activity

The antifungal activity of the dried hydroethanolic extracts was determined against *Candida albicans* ATCC 10231 and *Candida tropicalis* ATCC 13803 using the broth microdilution method. The microorganisms were first cultured in yeast extract, peptone and dextrose (YPD) broth until metabolically active cells were obtained. The fungal suspensions were then adjusted to a McFarland 0.5 standard.

To prepare the working solutions, 300 mg of each lyophilised extract was reconstituted in 1 mL of sterile distilled water. The samples were thoroughly homogenised before being dispensed into the microplates. The assay was carried out in sterile 96-well microplates. Serial two-fold dilutions were prepared from the initial solution of each extract in brain–heart infusion (BHI) broth. After completing the dilutions, 100 µL of each concentration was maintained per well, and 20 µL of the standardised fungal suspension was added, bringing the final volume to 120 µL.

The microplates were incubated at 37 °C for 24 h. At the end of this period, 20 µL of a 2,3,5-triphenyltetrazolium chloride (TTC) solution was added to each well. The plates were incubated again at 37 °C for 1–2 h, protected from light. The reduction in TTC and the consequent formation of a red colour were considered indicative of metabolic activity and yeast growth.

The minimum inhibitory concentration (MIC) was defined as the lowest concentration of the extract at which no red colour development was observed, corresponding to complete inhibition of fungal growth under the conditions tested. Fluconazole was used as the positive antifungal control. A growth control, consisting of the medium inoculated without extract; a sterility control, with medium without inoculum; and a negative control with sterile distilled water in place of the extract were also included. All determinations were carried out in triplicate.

### 4.6. In Vitro Antiproliferative Activity

Four tumour cell lines, HeLa (human cervical carcinoma, RRID: CVCL_0030), HepG2 (hepatocellular carcinoma, ATCC: HB-8065, RRID: CVCL_0027), HCT116 (human colon carcinoma, RRID: CVCL_0291), THJ29T (thyroid carcinoma, Applied Biological Materials Inc. (abm) Cat. No. T8254, RRID: CVCL_W922), and one non-tumour cell line NIH3T3 (mouse NIH/Swiss embryo fibroblasts, ATCC: CRL-1658, RRID: CVCL_0594) were cultured in Dulbecco’s Modified Eagle Medium (DMEM/F12) (Corning, Corning in Manassas, VA, USA) supplemented with 10% foetal bovine serum (FBS; Capricorn Scientific, Ebsdorfergrund, AleLes Ulis, France) and 1% penicillin/streptomycin (Thermo Fisher Scientific, Gibco, Miami, FL, USA). All cell lines were maintained in a humidified incubator at 37 °C with 5% CO_2_.

To evaluate the cytotoxic effect of the mushroom extracts, HeLa, THJ29T, and NIH3T3 cells were seeded at 1 × 10^4^ cells/well, whereas HepG2 and HCT116 were seeded at 2 × 10^4^ cells/well and 1.5× 10^4^ cells/well, respectively, in 96-well plates in 100 μL/well of DMEM/F12. The following day, the medium was replaced with 100 μL of seven serial dilutions of the mushroom extracts, in quadruplicate, ranging from 0.008 to 1 mg/mL for *S. luteus* and from 0.008 to 10 mg/mL for *A. bisporus* var. *brunnescens*, and plates were incubated for 72 h at 37 °C. Untreated cells maintained in complete medium served as controls, while wells containing medium without cells served as blanks. The extract stock solutions (250 mg/mL) were prepared in ultrapure water, stored refrigerated, and protected from light to preserve metabolite stability. After the incubation period, cell viability was assessed using the 3-(4,5-dimethylthiazol-2-yl)-2,5-diphenyltetrazolium bromide (MTT) assay, following the standard procedure provided by Sigma (St. Louis, MO, USA). Briefly, 10 μL of MTT solution (5 mg/mL) was added to each well. After 1 h of incubation at 37 °C, the medium was aspirated, and 50 μL of DMSO was added to each well to dissolve the formazan crystals. The mixture was gently agitated for 1 min at 300 rpm before measuring the absorbance at 570 nm using a Cytation 5 multi-mode detection system (BioTek, Winooski, VT, USA). To determine the concentration of the extract required to inhibit 50% of cell proliferation (IC_50_), dose–response curves were generated by nonlinear regression using a four-parameter logistic model with a variable Hill slope (log[inhibitor] vs. response) in GraphPad Prism version 10.0.0 for Windows (GraphPad Software, Boston, MA, USA). When the IC_50_ fell outside the experimentally tested concentration range, it was estimated by extrapolation from the fitted dose–response model. The untreated control group served as the basis for 100% cell proliferation. The therapeutic index (TI) was calculated as the ratio of the IC_50_ value in NIH3T3 non-tumour cells to that in each tumour cell line.

### 4.7. Anti-Inflammatory Activity

Prior to the anti-inflammatory assay, the cytotoxicity of both whole-fruiting-body extracts toward RAW 264.7 murine macrophages was evaluated using the MTT assay described in Section 4.6 to determine extract-specific IC_50_ values. The maximum concentrations tested were 1 mg/mL for *S. luteus* and 5 mg/mL for *A. bisporus* var. *brunnescens*. Serial dilutions were prepared in culture medium, and medium without extract was used as the blank. IC_50_ values were estimated from dose–response curves and are reported in Figure S1. Based on these results, concentrations of 0.075 and 0.15 mg/mL were selected as subcytotoxic concentrations for the anti-inflammatory assay for both extracts.

RAW 264.7 cells (ATCC TIB-71; RRID: CVCL_0493) were maintained in Dulbecco’s Modified Eagle Medium (DMEM; Corning, Manassas, VA, USA) containing 4.5 g/L glucose and L-glutamine and supplemented with 10% foetal bovine serum (FBS; Eurobio, Les Ulis, France) and 1% penicillin–streptomycin (Gibco, Thermo Fisher Scientific, Miami, FL, USA). Cells were cultured at 37 °C in a humidified atmosphere containing 5% CO_2_.

For the anti-inflammatory assay, cells were seeded in 24-well plates at a density of 3 × 10^5^ cells/well and incubated for 18–24 h. The culture medium was then replaced with serum-free DMEM containing dexamethasone (2 µg/mL) or either mushroom extract at 0.075 or 0.15 mg/mL. After 4 h of pretreatment, cells were stimulated with lipopolysaccharide (LPS; InvivoGen, San Diego, CA, USA) at a final concentration of 0.5 µg/mL and incubated for an additional 20 h.

Extracellular nitrite accumulation was quantified using the Griess assay. Briefly, 50 µL of culture supernatant was mixed with an equal volume of Griess reagent (Sigma-Aldrich, G4410; 50 mg/mL) and incubated for 10 min at room temperature in the dark. Absorbance was measured at 540 nm using a Cytation 5 multimode reader (BioTek). Nitrite concentrations were calculated using a 0.78–100 µM standard curve prepared with the Promega nitrite standard (G296A) after subtraction of the DMEM blank. Adherent cell biomass was assessed in parallel by crystal violet staining, with absorbance measured at 570 nm. Together with the previous MTT cytotoxicity assessment, this endpoint was used to determine whether changes in nitrite accumulation could be associated with loss of viable or adherent cells.

Experiments were performed using at least four independent biological replicates per group (n ≥ 4). Blank-corrected nitrite values under unstimulated conditions were predominantly below or close to the lowest calibration standard and were therefore summarised descriptively without inferential comparisons. Nitrite concentrations under LPS-stimulated conditions were analysed using a one-way mixed-effects model fitted by restricted maximum likelihood (REML), with treatment as a fixed effect and independent experiment as a matched random block. Sphericity was not assumed, and the Geisser–Greenhouse correction was applied, followed by Dunnett’s multiple-comparison test against the LPS-stimulated control. Paired Control and Control + LPS conditions were compared using an exact two-tailed Wilcoxon matched-pairs signed-rank test. All analyses were performed using GraphPad Prism version 11.0.1, and the complete statistical outputs are provided in the Supplementary Material.

### 4.8. Nematode Locomotion-Disrupting Activity

The nematode locomotion-disrupting activity of extracts obtained from *Suillus luteus* and *Agaricus bisporus* var. *brunnescens* was evaluated using synchronised L1 larvae of the wild-type *Caenorhabditis elegans* strain N2. Synchronised larvae were obtained by hypochlorite treatment of gravid hermaphrodites, followed by overnight hatching in sterile M9 buffer at 20 °C. Freshly hatched L1 larvae collected within 12–16 h after hatching were used to minimise developmental variability among assays.

As a food source, *Escherichia coli* OP50 was grown overnight in LB medium at 37 °C with agitation. Cells were collected by centrifugation and inactivated by UV irradiation for 30 min. The UV-killed bacterial suspension was washed three times with sterile M9 buffer by centrifugation at 4400 rpm for 5 min, then resuspended in M9 buffer. The suspension was adjusted to an OD600 of 0.4 before use.

For each assay, a master mix was prepared in sterile M9 buffer containing synchronised L1 larvae, UV-inactivated OP50, and cholesterol. The larval density was adjusted to deliver approximately 50 L1 larvae per well. OP50 was included at an OD600 of 0.4 in the master mix, corresponding to a final OD600 of approximately 0.2 in the assay wells, while cholesterol was added to a final concentration of 5 µg/mL. The suspension was gently but thoroughly mixed before dispensing to ensure homogeneous distribution of larvae and bacteria.

Extracts of *S. luteus* and *A. bisporus* var. *brunnescens* were tested independently at two predefined concentrations. *S. luteus* was evaluated at 0.125 and 50 mg/mL, whereas *A. bisporus* var. *brunnescens* was evaluated at 7.75 and 50 mg/mL. For both extracts, 50 mg/mL was the highest experimentally evaluable concentration without compromising nematode visualisation or automated tracking. The lower test concentrations were selected as practical values close to the corresponding IC_50_ values previously obtained in NIH3T3 non-tumour fibroblasts (0.11 mg/mL for *S. luteus* and 7.66 mg/mL for *A. bisporus* var. *brunnescens*; Table 5). These two concentrations were evaluated as discrete experimental conditions and were not used to generate a concentration–response curve.

Assays were performed in sterile flat-bottom 96-well microplates in a final volume of 150 µL per well. Briefly, 75 µL of each fungal extract dilution or control solution was first added to the corresponding wells, followed by 75 µL of the larva-containing master mix. Sterile M9 buffer was used as the negative control. A 2% (*w*/*v*) levamisole solution prepared from the commercial formulation Levalif^®^ 15% (levamisole hydrochloride, 150 mg/mL; Laboratorios LIFE C.A., Quito, Ecuador) was used as the positive locomotion-inhibition control.

To minimise evaporation during incubation, sterile M9 buffer was added to the spaces between wells and to the peripheral reservoirs of the microplates. Plates were incubated at 21 °C for up to 72 h under continuous gentle orbital agitation at 110 rpm to maintain larvae in suspension and ensure homogeneous exposure to the fungal extracts.

Nematode locomotion was recorded at the beginning of the assay and every 24 h thereafter for up to 72 h. Motility was quantified by video recording followed by automated analysis using the wrMTrck plugin in Fiji/ImageJ (ImageJ v1.54p; National Institutes of Health, Bethesda, MD, USA). Identical image-processing and tracking parameters were applied to all experimental conditions.

Locomotor performance was evaluated primarily using AvgSpeed and BLPS. AvgSpeed represents the mean translational speed of each tracked nematode over its recorded trajectory and was expressed in µm/s. BLPS (body lengths per second) represents locomotion speed normalised to the estimated body length of the tracked nematode and was expressed as body lengths/s.

Three videos, each obtained from a separate experimental well, were analysed for each condition. Measurements from all individual nematode tracks detected in each video were summarised as the median, and the resulting per-video median was treated as the experimental unit for statistical analysis (n = 3 independent wells per condition).

Because of the limited number of independent experimental observations and the inability to reliably assess normality, results were summarised as the median and range (minimum–maximum). Global differences among experimental conditions were evaluated using the Kruskal–Wallis rank-based test in Python 3.13.5 using SciPy 1.17.0. Statistical significance was defined as *p* < 0.05. Graphical representations were generated using Matplotlib 3.10.8. Each plotted point represents the median of all tracks detected in a single independent video; the central horizontal line represents the median of the three video-level observations; and whiskers extend from the minimum to the maximum value. Logarithmic scaling of the *y*-axis was used exclusively for graphical visualisation and did not alter the values used for statistical testing.

### 4.9. Statistical Analysis

The results are expressed as the mean ± standard deviation. For each physicochemical, mineral, bioactive compound, and in vitro chemical antioxidant variable, differences between the analysed parts were assessed using a one-way analysis of variance (ANOVA), with the mushroom organ as a fixed factor. The biological experimental unit was the independently collected or acquired lot. Three separate lots, each comprising 50 fruiting bodies, were evaluated per mushroom taxon. Analytical extractions and duplicate instrumental measurements performed for each lot were treated as technical replicates and averaged before statistical analysis. The normality of the residuals and the homogeneity of variances were assessed using the Shapiro–Wilk and Levene tests, respectively. Where the ANOVA indicated a significant effect, pairwise comparisons were carried out using Tukey’s test for significant differences, with a confidence level of α = 0.05.

## 5. Conclusions

A comparative analysis of *Suillus luteus* and *Agaricus bisporus* var. *brunnescens* showed that the species and their anatomical fractions have distinct chemical compositions and biological properties. Neither species had higher concentrations of all bioactive compounds. *A. bisporus* var. *brunnescens* had higher concentrations of vitamin C in the whole mushroom and the cap, as well as citric acid, tartaric acid, 4-hydroxybenzoic acid, syringic acid, and total phenols. In contrast, *S. luteus* was characterised by higher concentrations of total organic acids, malic acid, gallic acid, *m*-coumaric acid, and the variable defined as total anthocyanins, in addition to a higher vitamin C content in the stipe. The antioxidant capacity determined using the DPPH and ABTS assays was significantly higher in all fractions of *S. luteus*. In turn, *A. bisporus* var. *brunnescens* showed growth-inhibitory activity against all eight bacterial strains evaluated and exhibited its lowest MIC against *S. aureus* (3.9 mg/mL). *S. luteus* exhibited the greatest antiproliferative activity, with IC_50_ values ranging from 0.08 to 0.21 mg/mL, although tumour-cell selectivity was limited and the underlying cellular mechanisms remain undetermined. In the LPS-induced inflammation model, *S. luteus* significantly reduced nitrite accumulation by 43.3% and 64.6% at concentrations of 75 and 150 µg/mL, respectively, without decreasing cell viability or biomass. Further studies should assess additional inflammatory markers to better characterise this response. Although both extracts produced descriptive reductions in some locomotor parameters of *C. elegans* at the highest concentrations, the overall comparisons did not reach statistical significance. Overall, the crude hydroethanolic extracts exhibited distinct but complementary biological profiles: *S. luteus* showed the strongest antioxidant, antiproliferative and anti-inflammatory activities, whereas *A. bisporus* var. *brunnescens* displayed the broadest antibacterial spectrum under the experimental conditions evaluated. Because the extracts comprise complex mixtures of metabolites, the compounds responsible for these activities cannot yet be identified. Bioassay-guided fractionation, evaluation of purified constituents and their combinations, larger biological datasets, and additional microbial, cellular and mechanistic models will be required to confirm these findings and determine the constituents underlying the observed biological activities and preliminary locomotor trends.

## Figures and Tables

**Table 1:** Qualitative phytochemical profile of edible fungi.

Metabolites	*Suillus luteus*			*Agaricus bisporus* var. *brunnescens*		
	Whole	Cap	Stipe	Whole	Cap	Stipe
Steroids	-	-	-	-	-	-
Terpenoids	-	-	-	-	-	-
Phenols	+	+	+	+	+	+
Tannins	+	+	+	+	+	+
Alkaloids	-	-	-	-	-	-
Flavonoids	-	-	-	-	-	-
Saponins	-	-	-	-	-	-
Acetogenins	-	-	-	+	+	+

Note: -, Negative test result; +, Positive test result.

**Table 2:** Average physicochemical characteristics and mineral composition of edible mushrooms.

	*Suillus luteus*			*Agaricus bisporus* var. *brunnescens*					
Characteristics	Whole	Cap	Stipe	Whole	Cap	Stipe	A_Whole_	A_Cap_	A_Stipe_
pH	5.7 ± 0.0 ^a^	5.6 ± 0.0 ^a^	5.8 ± 0.0 ^a^	6.5 ± 0.0 ^a^	6.6 ± 0.0 ^a^	6.4 ± 0.0 ^a^	***	ns	ns
SS (°Brix)	1.4 ± 0.0 ^a^	1.1 ± 0.1 ^a^	2.2 ± 0.1 ^a^	1.2 ± 0.1 ^b^	1.1 ± 0.1 ^a^	1.1 ± 0.1 ^a^	*	ns	***
TA (%)	0.1 ± 0.0 ^a^	0.1 ± 0.0 ^a^	0.1 ± 0.0 ^a^	0.1 ± 0.1 ^a^	0.1 ± 0.0 ^a^	0.1 ± 0.0 ^a^	ns	ns	ns
Humidity (%)	94.0 ± 0.1 ^a^	94.2 ± 0.1 ^a^	92.1 ± 0.6 ^a^	89.9 ± 0.1 ^a^	89.7 ± 0.6 ^a^	92.2 ± 0.1 ^b^	***	***	ns
Ash (%)	0.5 ± 0.1 ^a^	0.5 ± 0.1 ^a^	0.1 ± 0.0 ^b^	1.0 ± 0.0 ^b^	1.0 ± 0.1 ^b^	1.2 ± 0.1 ^b^	***	***	***
Ca (mg/100 g DW)	51.9 0.0 ^a^	51.5 0.0 ^a^	58.4 0.0 ^a^	32.4 0.0 ^a^	70.8 0.0 ^a^	71.0 0.0 ^a^	***	***	ns
Fe (mg/100 g DW)	23.1 0.0 ^a^	22.9 0.2 ^a^	23.1 0.0 ^a^	23.0 0.2 ^a^	22.9 0.3 ^a^	22.9 0.2 ^a^	ns	ns	ns
Na (mg/100 g DW)	42.0 1.2 ^b^	58.4 1.9 ^a^	59.4 1.9 ^a^	80.9 1.3 ^a^	70.5 1.1 ^c^	75.5 1.8 ^b^	***	***	***
K (mg/100 g DW)	3269.1 7.1 ^b^	3347.6 4.0 ^b^	3839.4 12.1 ^a^	3980.4 31.0 ^c^	4899.9 14.1 ^a^	4589.4 24.0 ^b^	***	***	***

Note: Values indicate average ± standard deviation. SS, soluble solids; TA, titratable acidity; Ca, calcium; Fe, iron; Na, sodium; K, potassium. The lowercase letters next to the deviations indicate significant differences among parts of the fungus. A_Whole_ indicates significant differences when comparing the two complete fungi. A_Cap_ indicates significant differences when comparing the caps of the two fungi under study. A_Stipe_ indicates significant differences between the stipes of the two fungi under study. ns, not significant; *, *p* < 0.05; *** *p* < 0.005.

**Table 3:** Average bioactive compounds of edible fungi.

Concentration (mg/100 g DW)	*Suillus luteus*			*Agaricus bisporus* var. *brunnescens*					
	Whole	Cap	Stipe	Whole	Cap	Stipe	A_Whole_	A_Cap_	A_Stipe_
Vitamin C	14.2 ± 0.9 ^b^	15.2 ± 0.0 ^b^	19.0 ± 2.4 ^a^	46.7 ± 1.3 ^a^	50.2 ± 0.1 ^b^	16.1 ± 3.8 ^c^	***	***	***
Citric acid	54.6 ± 0.7 ^c^	127.4 ± 9.2 ^b^	173.7 ± 1.6 ^a^	1755.2 ± 10.2 ^b^	1526.2 ± 10.2 ^b^	1722.5 ± 38.9 ^a^	***	***	***
Malic acid	43,249.5 ± 17.7 ^b^	38,355.7 ± 22.9 ^c^	51,639.3 ± 10.7 ^a^	7181.0 ± 10.1 ^a^	9975.5 ± 13.2 ^b^	6672.5 ± 87.3 ^c^	***	***	***
Tartaric acid	141.9 ± 2.0 ^b^	125.9 ± 0.7 ^c^	161.6 ± 2.5 ^a^	4082.6 ± 1.3 ^a^	3746.6 ± 8.4 ^b^	3537.0 ± 20.3 ^c^	*	***	***
Total organic acids	43,446.0 ± 17.6 ^b^	38,609.0 ± 23.8 ^c^	51,974.6 ± 10.8 ^a^	13,018.8 ± 11.2 ^a^	15,248.3 ± 75.2 ^a^	11,932.0 ± 12.8 ^b^	***	***	***
Total anthocyanin	1476.3 ± 19.3 ^b^	2963.3 ± 38.8 ^a^	2988.9 ± 28.7 ^a^	919.7 ± 32.6 ^a^	1544.9 ± 32.1 ^b^	1368.2 ± 12.4 ^c^	**	***	***
Gallic acid	15.1 ± 0.1 ^c^	18.0 ± 0.3 ^b^	24.5 ± 1.6 ^a^	9.8 ± 0.1 ^a^	9.1 ± 1.0 ^a^	6.3 ± 1.2 ^b^	***	***	***
4-Hydroxybenzoic acid	370.7 ± 2.8 ^a^	362.2 ± 59.7 ^a^	395.5 ± 25.8 ^a^	1575.5 ± 4.1 ^a^	1872.0 ± 23.3 ^b^	1481.6 ± 44.8 ^c^	***	***	***
*m*-Coumaric acid	1065.8 ± 11.7 ^b^	943.7 ± 88.7 ^c^	1160.8 ± 10.4 ^a^	81.9 ± 1.5 ^a^	93.7 ± 5.6 ^a^	49.6 ± 1.9 ^b^	***	***	***
Syringic acid	63.3 ± 1.6 ^a^	34.0 ± 0.1 ^c^	53.7 ± 0.4 ^b^	192.1 ± 8.7 ^a^	178.0 ± 3.6 ^a^	148.8 ± 1.8 ^b^	***	***	***
Total phenolics	1514.9 ± 14.5 ^b^	1358.0 ± 18.2 ^c^	1634.5 ± 9.7 ^a^	1859.3 ± 7.6 ^b^	2152.8 ± 1.9 ^a^	1685.9 ± 7.8 ^c^	***	***	*

Note: Values indicate average ± standard deviation. The lowercase letters next to the deviations indicate significant differences among parts of the fungus. A_Whole_ indicates significant differences when comparing the two complete fungi. A_Cap_ indicates significant differences when comparing the caps of the two fungi under study. A_Stipe_ indicates significant differences between the stipes of the two fungi under study. *, *p* < 0.05; **, *p* < 0.01; *** *p* < 0.005.

**Table 4:** Average in vitro antioxidant activity of edible fungi.

	*Suillus luteus*			*Agaricus bisporus* var. *brunnescens*					
Activity (mmol TE/100 g DW)	Whole	Cap	Stipe	Whole	Cap	Stipe	A_Whole_	A_Cap_	A_Stipe_
Antioxidant activity by DPPH	3.3 ± 0.1 ^a^	3.2 ± 0.0 ^a^	3.2 ± 0.0 ^a^	2.7 ± 0.1 ^a^	3.0 ± 0.1 ^a^	2.3 ± 0.1 ^b^	***	***	***
Antioxidant activity by ABTS	5.9 ± 0.1 ^a^	5.5 ± 0.0 ^b^	4.9 ± 0.0 ^c^	4.2 ± 0.1 ^b^	4.8 ± 0.1 ^a^	4.1 ± 0.1 ^c^	***	***	***

Note: Values indicate average ± standard deviation. The lowercase letters next to the deviations indicate significant differences among parts of the fungus. A_Whole_ indicates significant differences when comparing the two complete fungi. A_Cap_ indicates significant differences when comparing the caps of the two fungi under study. A_Stipe_ indicates significant differences between the stems of the two fungi under study. *** *p* < 0.005.

**Table 5:** Minimum inhibitory concentration of the hydroethanolic mushroom extracts (mg/mL).

Microorganisms	*Suillus luteus*	*Agaricus bisporus* var. *brunnescens*
*E. coli* ATCC 8739	62.5	124.9
*P. aeruginosa* ATCC 9027	-	124.9
*S. aureus* ATCC 6538P	62.5	3.9
*S. mutans* ATCC 25175	62.5	31.3
*P. fluorescens* ATCC 13525	-	125.0
*S. epidermidis* ATCC 12228	-	15.6
*P. putida* ATCC 31483	-	62.5
*E. tarda* ATCC 15947	-	31.3
*C. albicans* ATCC 10231	-	-
*C. tropicalis* ATCC 13803	-	-

Note: -, inactive at the tested concentrations.

**Table 6:** IC_50_ values (mg/mL) and therapeutic index (TI) of mushroom extracts in tumour and non-tumour cell lines.

	HeLa		HCT116		THJ29T		HepG2		NIH3T3
	IC_50_	TI	IC_50_	TI	IC_50_	TI	IC_50_	TI	IC_50_
*Suillus luteus*	0.21 ± 0.02	0.52	0.19 ± 0.02	0.58	0.08 ± 0.01	1.38	0.18 ± 0.02	0.61	0.11 ± 0.01
*Agaricus bisporus* var. *brunnescens*	7.21 ± 0.91	1.06	5.76 ± 0.41	1.33	6.46 ± 0.80	1.19	15.70 ± 1.20	0.49	7.66 ± 1.24

## Data Availability

The original contributions presented in this study are included in the article. Further inquiries can be directed to the corresponding author.

## Supplementary Materials

The following supporting information can be downloaded at: https://www.mdpi.com/article/10.3390/ph19101571/s1, Figure S1: Concentration-dependent effects of *S. luteus* and *A. bisporus* extracts on MTT metabolic activity in RAW 264.7 macrophages following 72 h of exposure; Table S1: Statistical analysis of extracellular nitrite concentrations under LPS-stimulated conditions; Table S2: Statistical comparison of extracellular nitrite concentrations between unstimulated and LPS-stimulated controls.
