# Supplementary material for: Exploring Chemical Diversity Alongside Antioxidant, Antimicrobial, Antiproliferative, Anti-Inflammatory, and Caenorhabditis elegans Locomotor Responses in Whole, Caps and Stipes of Suillus luteus and Agaricus bisporus
Source: Pharmaceuticals (Basel). 2026 Oct 3;19(10):1571. doi: 10.3390/ph19101571 (PMC13708346; doi:10.3390/ph19101571)
Supplement: Supplementary file 1 [file pharmaceuticals-19-01571-s001.zip › pharmaceuticals-4577979-supplementary/Suplementar-HongoPino.pdf]

Article

Exploring Chemical Diversity Alongside Antioxidant, Antimicrobial, Antiproliferative, Anti-Inflammatory, and *Caenorhabditis elegans* Locomotor Responses in Whole, Caps and Stipes of *Suillus luteus* and *Agaricus bisporus*

Elena Coyago-Cruz [1,*], Juan A. Puente-Pineda [2], Rebeca Gonzalez-Pastor [2], Julieth Soledispa [1], Angélica Geovanna Zea Cobos [3], Oscar Carrillo-Naranjo [2], Carlos Barba-Ostria [4,5], Jorge Heredia-Moya [2] and Gina Tafur [6]

1 Carrera de Ingeniería en Biotecnología, Universidad Politécnica Salesiana, Sede Quito, Campus El Girón, Av. 12 de Octubre N2422 y Wilson, Quito 170143, Ecuador
2 Centro de Investigación Biomédica (CENBIO), Facultad de Ciencias de la Salud Eugenio Espejo, Universidad UTE, Quito 170527, Ecuador
3 Grupo de Innovación Educativa, Seguridad y Soberanía Alimentaria (GIE-SYSA), Universidad Politécnica Salesiana, Sede Cuenca, Av. Turuhuayco 3-69 y Calle Vieja, Cuenca 010150, Ecuador
4 Escuela de Medicina, Colegio de Ciencias de la Salud Quito, Universidad San Francisco de Quito (USFQ), Quito 170901, Ecuador
5 Instituto de Microbiología, Universidad San Francisco de Quito (USFQ), Quito 170901, Ecuador
6 Carrera de Ingeniería Agropecuaria, Universidad Politécnica Salesiana, Sede Quito, Campus Cayambe, Av. Natalia Jarrín N3-85 y 9 de Octubre, Cayambe 170501, Ecuador
* Correspondence: ecoyagoc@ups.edu.ec; Tel.:+593-995129321

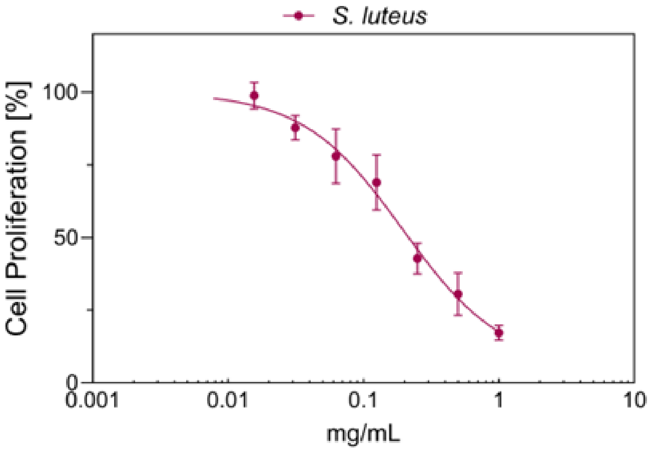

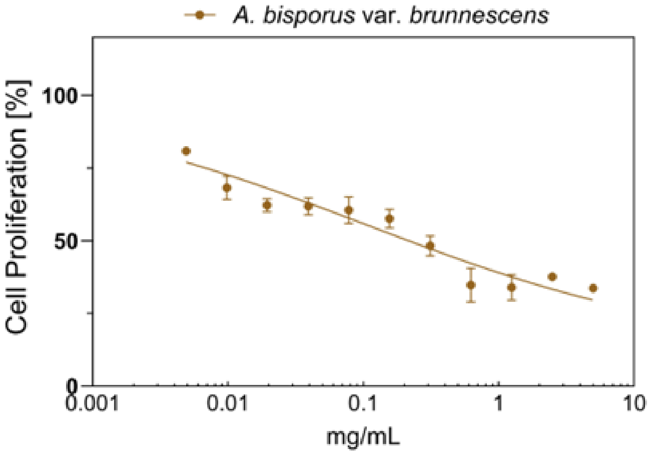

Figure S1. Concentration-dependent effects of *S. luteus* and *A. bisporus* extracts on MTT metabolic activity in RAW 264.7 macrophages following 72 h of exposure. IC_{50} values were estimated by nonlinear regression. Data are presented as mean ± SD.

Table S1. Statistical analysis of extracellular nitrite concentrations under LPS-stimulated conditions.

Comparison	Paired experiments (n)	Mean difference (µM)	SE	q	df	Adjusted p value	Significance
Control + LPS vs. DEX + LPS	6	8.002	0.9194	8.703	5	0.0012	**
Control + LPS vs. *S. luteus* 75 µg/mL + LPS	6	8.947	1.080	8.285	5	0.0015	**
Control + LPS vs. *S. luteus* 150 µg/mL + LPS	6	16.20	0.9555	16.96	5	<0.0001	****
Control + LPS vs. *A. bisporus* var. *brunnescens* 75 µg/mL + LPS	4	2.530	0.9212	2.746	3	0.1934	ns
Control + LPS vs. *A. bisporus* var. *brunnescens* 150 µg/mL + LPS	4	4.178	1.252	3.337	3	0.1245	ns

Note: Nitrite concentrations were analyzed using a one-way mixed-effects model fitted by restricted maximum likelihood, with treatment as a fixed effect and independent experiment as a matched random block. The Geisser–Greenhouse correction was applied because sphericity was not assumed. The overall treatment effect was statistically significant, $F(0.5872, 2.466) = 101.2$, $p = 0.0049$, $\varepsilon = 0.1174$. Dunnett's test compared each treatment with the LPS-stimulated control. Mean differences were calculated as Control + LPS minus treatment + LPS; positive values therefore indicate lower nitrite accumulation following treatment. Reported *p* values are multiplicity-adjusted. ns, $p \geq 0.05$; **, $p < 0.01$; ****, $p < 0.0001$. DEX, dexamethasone; LPS, lipopolysaccharide.

Table S2. Statistical comparison of extracellular nitrite concentrations between unstimulated and LPS-stimulated controls.

Comparison	Paired experiments (n)	Median paired difference (µM)	Exact confidence interval	W	Exact two-tailed p value	Significance
Control + LPS vs. Control	8	34.37	17.31–51.53 (99.22% CI)	36.00	0.0078	††

Note: Paired Control and Control + LPS values obtained within the same independent experiments were compared using an exact two-tailed Wilcoxon matched-pairs signed-rank test. Differences were calculated as Control + LPS minus Control. LPS significantly increased extracellular nitrite accumulation, with a median paired difference of 34.37 µM, $W = 36.00$ and exact $p = 0.0078$. Prism reported an exact 99.22% confidence interval of 17.31–51.53 µM, representing the closest attainable exact interval to the requested 95% confidence level for eight paired observations. ††, $p < 0.01$.
